# Cerebrospinal fluid profile of NPTX2 supports role of Alzheimer’s disease-related inhibitory circuit dysfunction in adults with Down syndrome

**DOI:** 10.1186/s13024-020-00398-0

**Published:** 2020-08-17

**Authors:** Olivia Belbin, Mei-Fang Xiao, Desheng Xu, Maria Carmona-Iragui, Jordi Pegueroles, Bessy Benejam, Laura Videla, Susana Fernández, Isabel Barroeta, Raúl Nuñez-Llaves, Victor Montal, Eduard Vilaplana, Miren Altuna, Jordi Clarimón, Daniel Alcolea, Rafael Blesa, Alberto Lleó, Paul F. Worley, Juan Fortea

**Affiliations:** 1 Memory Unit and Biomedical Research Institute Sant Pau (IIB Sant Pau), Neurology Department, Hospital de la Santa Creu i Sant Pau, 08025 Barcelona, Spain; 2 Centro de Investigación Biomédica en Red sobre Enfermedades Neurodegenerativas (CIBERNED), 28031 Madrid, Spain; 3 Solomon H. Snyder Department of Neuroscience, Johns Hopkins University School of Medicine, Baltimore, MD 21205 USA; 4 Barcelona Down Medical Center, Fundació Catalana Síndrome de Down, Barcelona, Spain; 5 Department of Neurology, Johns Hopkins University School of Medicine, Baltimore, MD 21205 USA

**Keywords:** Neuronal Pentraxin-2, Alzheimer’s disease, Down syndrome, Inhibitory circuits, Cerebrospinal fluid, Biomarker, GluA4, Cortical atrophy, Glucose metabolism

## Abstract

**Background:**

Alzheimer’s disease (AD) is the major cause of death in adults with Down syndrome (DS). There is an urgent need for objective markers of AD in the DS population to improve early diagnosis and monitor disease progression. NPTX2 has recently emerged as a promising cerebrospinal fluid (CSF) biomarker of Alzheimer-related inhibitory circuit dysfunction in sporadic AD patients. The objective of this study was to evaluate NPTX2 in the CSF of adults with DS and to explore the relationship of NPTX2 to CSF levels of the PV interneuron receptor, GluA4, and existing AD biomarkers (CSF and neuroimaging).

**Methods:**

This is a cross-sectional, retrospective study of adults with DS with asymptomatic AD (aDS, *n* = 49), prodromal AD (pDS, *n* = 18) and AD dementia (dDS, *n* = 27). Non-trisomic controls (*n* = 34) and patients with sporadic AD dementia (sAD, *n* = 40) were included for comparison. We compared group differences in CSF NPTX2 according to clinical diagnosis and degree of intellectual disability. We determined the relationship of CSF NPTX2 levels to age, cognitive performance (CAMCOG, free and cued selective reminding, semantic verbal fluency), CSF levels of a PV-interneuron marker (GluA4) and core AD biomarkers; CSF Aβ_1–42_, CSF t-tau, cortical atrophy (magnetic resonance imaging) and glucose metabolism ([^18^F]-fluorodeoxyglucose positron emission tomography).

**Results:**

Compared to controls, mean CSF NPTX2 levels were lower in DS at all AD stages; aDS (0.6-fold, adj.*p* < 0.0001), pDS (0.5-fold, adj.*p* < 0.0001) and dDS (0.3-fold, adj.*p* < 0.0001). This reduction was similar to that observed in sporadic AD (0.5-fold, adj.*p* < 0.0001). CSF NPTX2 levels were not associated with age (*p* = 0.6), intellectual disability (*p* = 0.7) or cognitive performance (all *p* > 0.07). Low CSF NPTX2 levels were associated with low GluA4 in all clinical groups; controls (*r*^2^ = 0.2, *p* = 0.003), adults with DS (*r*^2^ = 0.4, *p* < 0.0001) and sporadic AD (*r*^2^ = 0.4, *p* < 0.0001). In adults with DS, low CSF NPTX2 levels were associated with low CSF Aβ_1–42_ (*r*^2^ > 0.3, *p* < 0.006), low CSF t-tau (*r*^2^ > 0.3, *p* < 0.001), increased cortical atrophy (*p* < 0.05) and reduced glucose metabolism (*p* < 0.05).

**Conclusions:**

Low levels of CSF NPTX2, a protein implicated in inhibitory circuit function, is common to sporadic and genetic forms of AD. CSF NPTX2 represents a promising CSF surrogate marker of early AD-related changes in adults with DS.

## Background

Down syndrome (DS) has been conceptualized by the International Working Group for Alzheimer’s disease (AD) as a genetically determined form of AD [1] due to the triplication of the gene encoding amyloid precursor protein (APP), among others [2]. AD in DS has a long preclinical phase and a predictable sequence of changes in biomarkers that is strikingly similar (both in order and temporality) to that described in autosomal dominant AD [3, 4]. The similar patterns of amyloid deposition, atrophy and hypometabolism indicate that AD in DS targets the same cortical regions as those affected in the sporadic and autosomal dominant forms [5]. This is supported by neuropathological studies, which show that DS brains from donors who arrive to autopsy in the 4th decade present AD pathology [6]. The cumulative incidence of AD dementia exceeds 90% in the seventh decade with a median age-of-onset of 55 years [7, 8], and as such, AD is now the leading cause of death in adults with DS [9]. While neuropsychological tests are able to detect cognitive decline in this population, the intellectual disability associated with the syndrome can complicate an AD diagnosis. Thus, there is an urgent need for objective markers of AD in adults with DS to improve early diagnosis, monitor disease progression and assess response to treatment.

Accumulating evidence suggests that early dysfunction of inhibitory interneuronal circuits contributes to cognitive abnormalities in AD decades before clinical disease onset [10]. It follows that a surrogate marker of inhibitory circuit dysfunction could be a valuable biomarker of early AD pathogenesis. In support of this, recent studies have shown that neuronal pentraxin-2 (NPTX2), a protein that accumulates at excitatory synapses onto GABA-ergic parvalbumin (PV) interneurons [11], is down-regulated in pathologically confirmed sporadic AD and adult trisomy 21 brains [12]. At the excitatory synapse on PV interneurons, NPTX2 has been shown to mediate activity-dependent increases of the AMPA receptor subunit, GluA4, which is selectively expressed in PV interneurons [11]. This action of NPTX2 enhances PV interneuron activity and thereby inhibitory circuit function important for homeostatic maintenance of excitation/inhibition balance and for circuit rhythmicity essential for memory [13, 14]. GluA4 is also down-regulated in AD brains in a coordinated manner with NPTX2 [12], consistent with a disruption of PV interneuron function. The reduction in brain NPTX2 expression is also reflected in the CSF and a recent study has shown that the ratio of CSF NPTX2 to CSF t-tau (neurodegeneration marker) was the best CSF predictor of cognitive decline in sporadic AD [15].

Based on the aforementioned data, we hypothesized that, as a genetic form of AD, DS CSF will have reduced NPTX2 and correlated GluA4 levels, reflecting PV interneuron dysfunction, thus representing a potential surrogate marker of early AD pathophysiology in adults with DS. To test this hypothesis, we have evaluated CSF levels of NPTX2 as a surrogate marker of inhibitory circuit dysfunction using the Down Alzheimer Barcelona Neuroimaging Initiative (DABNI) cohort, the largest collection worldwide of CSF and neuroimaging data collected from adults with DS (*n* = 94). We have included non-trisomic population controls (*n* = 34) and sporadic AD patients (*n* = 40) from the Sant Pau Initiative for Neurodegeneration (SPIN) cohort for comparison.

## Methods

### Study design

This is a single-center cross-sectional study of cognitively normal non-trisomic individuals, adults with DS and sporadic AD patients. Controls and sporadic AD patients were retrospectively selected from the Sant Pau Initiative for Neurodegeneration (SPIN) cohort (Memory Unit, Hospital de la Santa Creu i Sant Pau, Barcelona, Spain, [16]). Controls were without cognitive or neurological disorders and normal levels of core AD CSF biomarkers (Aβ_1–42_, t-tau and p-tau), determined according to our internal cut-offs [17]. AD patients were clinically diagnosed with dementia due to AD [18]. Adults with DS were recruited from the Down Alzheimer Barcelona Neuroimaging Initiative, a prospective longitudinal cohort, linked to a population-based health plan in Catalonia, Spain, led by the Catalan Foundation for Down Syndrome and Hospital de la Santa Creu i Sant Pau, Barcelona, Spain [19]. Inclusion criteria for participation in the study required that participants were over 18 years of age, that they received a comprehensive neurological and neuropsychological evaluation and underwent a lumbar puncture to assess AD biomarkers levels (CSF Aβ_1–42_, t-tau and p-tau) [17]. As in previous studies [19–21], participants with DS were classified by neurologists and neuropsychologists, masked to biomarker data in a consensus meeting into the following groups: asymptomatic AD (aDS; no clinical or neuropsychological suspicion of symptomatic AD), prodromal AD (pDS; suspicion of cognitive decline due to AD, but symptoms did not fulfill criteria for dementia) and AD dementia (dDS; full blown dementia).

### Neuropsychological assessment

The level of intellectual disability was categorized according to the Diagnostic and Statistical Manual of Mental Disorders, Fifth Edition as mild, moderate, severe, or profound intellectual disability, based on carers’ reports of the individuals’ best-ever level of functioning and the Kaufmann Brief Intelligence Test [22]. Neurological and neuropsychological examination of individuals with DS, as previously described [15], included a semi-structured health questionnaire (Cambridge Examination for Mental Disorders of Older People with DS and others with intellectual disabilities) [23] and a neuropsychological battery including the Cambridge Cognition Examination (CAMCOG) adapted for intellectual disabilities in individuals with DS limited to mild or moderate intellectual disability. Declarative verbal memory was evaluated by the Free and Cued Selective Reminding Test (FCSRT) as previously described [24].

### Karyotyping

DNA samples extracted from blood were subjected to high-density single nucleotide polymorphism genotyping with the Illumina Infinium Global Screening Array (Illumina, San Diego, CA, USA). Visualization and confirmation of chromosome 21 aneuploidy was done through the Genome Viewer tool within Genome Studio version 2.0 (Illumina). A shift towards 66 and 33% in the B allele frequency, and an increase from approximately 0 to 0.25 in the log R ratio across all single nucleotide polymorphisms contained within chromosome 21 was used as an indicator of trisomy [25]. All adults with DS included in the study had confirmed trisomy of chromosome 21.

### CSF collection and biomarker assessment

CSF samples were collected following international consensus recommendations [26] as previously described [27]. Samples had been previously stored at − 80 °C and had not been thawed prior to analysis. Commercially available automated immunoassays were used to determine levels of CSF Aβ_1–42_ (Lumipulse Aβ_1–42_, Fujirebio-Europe, Belgium) total tau (Lumipulse G Total Tau, Fujirebio-Europe) and tau phosphorylated at threonine residue 181 (Lumipulse G PTAU 181, Fujirebio-Europe) in the DABNI cohort and SPIN controls [17]. An in-house enzyme-linked immunosorbent assay targeting the N-terminal fragment encoding amino acid 1 to 201 of NPTX2 was performed in the Baltimore Laboratory as previously described [12].

### Targeted liquid chromatography mass spectrometry

We monitored a set of 3 proteotypic peptides corresponding to GluA4 by Selected Reaction Monitoring (SRM) as previously described [28]. Briefly, we digested individual CSF samples overnight and spiked isotopically-labeled peptides (Pepotech SRM custom peptides, grade 2, Thermo Fisher Scientific) into each sample. We analyzed an equivalent of 5ul of each sample in a randomized order over a 120-min gradient (0–35% ACN + 0.1%FA) in SRM mode using a triple quadrupole-Qtrap mass spectrometer (5500 QTrap, Sciex, Massachussetts) coupled to a nano-LC chromatography column (300 ul/min, 25-cm C18 column, 75 um I.d., 2 um particle size). We ran BSA technical controls between each sample. We visualized and analyzed transitions using Skyline 3.5. Injection of a pool of all the samples over the duration of the mass spectrometric measurements and monitoring the peak area of the standard peptides confirmed the stability of all peptides over the course of the experiment. We processed the SRM transitions using the dataProcess function of MSstats v3.5 package in R [29] and removed transitions with between run interference (betweenRunInterferenceScore< 0.8). Censored missing values were samples with log base-2 endogenous intensities under the cut-off designated by the MSstats package (10.35). We used the EqualizeMedians function to normalize the transitions and Tukey’s Median Polish to generate a summarized value.

### Magnetic resonance imaging (MRI) acquisition and analysis

A high-resolution three-dimensional structural dataset was acquired in a 3 T MRI scanner (Philips 3.T X Series ACHIEVA) with the following parameters: T1-weighted magnetization-prepared rapid gradient-echo, repetition time 8.1 msec, echo time 3.7 msec, 160 slices, matrix size 240 × 234; slice thickness 1 mm, voxel size 0.94 × 0.94 × 1 mm. Cortical thickness reconstruction was performed with Freesurfer package v6 (http://surfer.nmr.mgh.harvard.edu) using a procedure that has been described in detail elsewhere [30], as previously reported [28, 29]. A Gaussian kernel of 15 mm full width at half maximum (FWHM) was applied to the subjects’ cortical thickness maps before further analyses as it is customary in surface-based analyses. From the 75 adults with DS who had available MRI, 14 were excluded due to suboptimal image quality, which included subtle movement artifacts, poor signal to noise ratio, and gradient artifacts. The breakdown of diagnosis in these individuals was as follows: aDS *n* = 38, pDS *n* = 15, dDS *n* = 8.

### FDG-PET acquisition and analysis

From the remaining 61 adults with DS with MRI, 42 underwent [18F]-fluorodeoxyglucose (FDG) positron emission tomography (PET) scan on an integrated PET-TC system (Philips Gemini TF). The breakdown of diagnosis in these individuals was as follows: aDS *n* = 28, pDS *n* = 10, dDS *n* = 4. Overnight fasting subjects’ plasma glucose was measured and confirmed to be < 180 mg/dL prior to starting the scanning procedure. Data were acquired 50 min after the injection of 259 MBq/ml (7 mCi) of [^18^F]-FDG. After computerized tomography data was obtained, brain PET was acquired. The reconstruction method was iterative (LOR RAMBLA, 3 iterations and 33 subsets) with a 128 × 128 image size, 2 mm pixel size and 2 mm pixel slice thickness. [^18^F]-FDG-PET images were intensity-scaled by the reference pons-vermis region [31], spatially normalized to the Freesurfer anatomical space using a PET surfer approach [32] and then smoothed with a 10 mm FWHM kernel in order to obtain similar relative smoothing FDG maps as compared with cortical thickness maps [33, 34]. With this surface-based approach, the reliability of PET effects improves and the inter-individual variance is reduced [35]. All resulting images were visually inspected to check for possible registration errors.

### Statistical analysis

Statistical analyses were performed in R version 3.4.3 [36]. Group differences were compared using One-way Analysis of Variance (ANOVA) with post hoc Tukey tests adjusted for multiple testing using the Bonferonni-Hochberg method. Robust logistic regressions were performed using the lm function included in the “robustbase” package that uses MM-type regression estimators to compute robustness weights in the fitting process. All reported *r*^2^ values were adjusted for the number of predictors in the model. Where residuals deviated from a Gaussian distribution (Shapiro-Wilk *p* < 0.05), square root transformed values were used. Outliers were excluded using the 3 × IQR rule. For the neuroimaging analyses we performed Monte-Carlo simulation with 10,000 repeats (family wise error [FWE] correction at *p* < 0.05). Due to the small size of each clinical group with available neuroimaging, analyses were performed on the complete DS dataset only. The figures show only those results that survived FWE correction. Alpha was set at 0.05 for global analyses and 0.01 for post-hoc analyses.

## Results

The Table 1 shows the demographic and clinical data for the samples included in the study. The mean age was 52 years (standard deviation 15) across the whole study (*n* = 168). Compared to controls the mean age was higher in the sporadic AD group (+ 19 years, 95% CI 13 to 25, adj.*p* < 0.0001), lower in the aDS group (− 14 years, 95% CI 8 to 19; adj.*p* < 0.0001) and comparable in pDS and dDS (both adj.*p* > 0.9). As would be expected for an age-related disease such as AD, mean age was lower in aDS than pDS (− 14 years, 95% CI − 7 to − 21; adj.*p* < 0.0001) and dDS (− 15 years, 95% CI − 9 to − 21; adj.*p* < 0.0001). The proportion of males:females included in the study was 46:54 and was comparable across diagnostic groups (*p* = 0.09). The proportion of individuals with severe/profound intellectual disability was comparable across AD stages (*p* = 0.7). All cognitive measures were lower in sAD compared to controls (all adj.*p* < 0.0001) and in dDS compared to aDS (all adj.*p* < 0.0001). CSF biomarker levels were as previously reported [17]. Briefly, compared to controls mean Aβ_1–42_ levels were lower in all groups (all adj.*p* < 0.0001) and p-tau and t-tau levels were higher in sAD, pDS and dDS (all adj.*p* < 0.0001).

**Table 1:** Demographics and clinical data for study participants

Group	Control	sAD	aDS	pDS	dDS
**N**	**34**	**40**	**49**	**18**	**27**
**Mean age-at-collection (years)**	**51**	**70**	**37**	**52**	**52**
SD (range)	13 (24–74)	7 (52–83)	10 (22–57)	4 (45–60)	5 (42–62)
**Sex (% Female)**	**68%**	**65%**	**47%**	**44%**	**42%**
**Intellectual Disability**^a^	**None**	**None**	**80%;20%**	**78%;22%**	**67%;33%**
**FCSRT Immediate Total Recall**	**34**	**13**	**35**	**19**	**15**
SD (range), n	3 (34–48), 40	10 (0–36), 30	2 (30–36), 33	11 (0–36), 11	7 (0–32), 13
**Semantic Verbal Fluency Test**	**22**	**9**	**10**	**8**	**7**
SD (range), n	5 (14–33), 34	4 (3–15), 30	3 (3–18), 37	2 (4–13), 12	4 (1–12), 15
**CAMCOG**	**N/A**	**N/A**	**79**	**67**	**54**
SD (range), n	N/A	N/A	12 (51–96), 36	12 (41–80), 10	22 (2–87), 11
**Mean CSF Aβ**_**1–42**_ **(pg/ml)**	**1359**	**492**	**1089**	**489**	**504**
SD (range)	284 (1000–2034)	107 (275–690)	474 (302–2335)	146 (235–865)	160 (235–765)
**Mean CSF t-tau (pg/ml)**	**249**	**781**	**342**	**972**	**1095**
SD (range)	63 (157–407)	423 (229–2110)	248 (86–1394)	658 (118–2565)	653 (212–3150)
**Mean CSF p-tau (pg/ml)**	**37**	**137**	**44**	**173**	**173**
SD (range)	9 (22–55)	62 (55–351)	47 (10–277)	128 (22–564)	92 (31–354)

^a^Proportion of cases with mild/moderate vs severe/profound intellectual disability

### CSF NPTX2 levels are similarly reduced in DS and sporadic AD

**Fig. 1:**
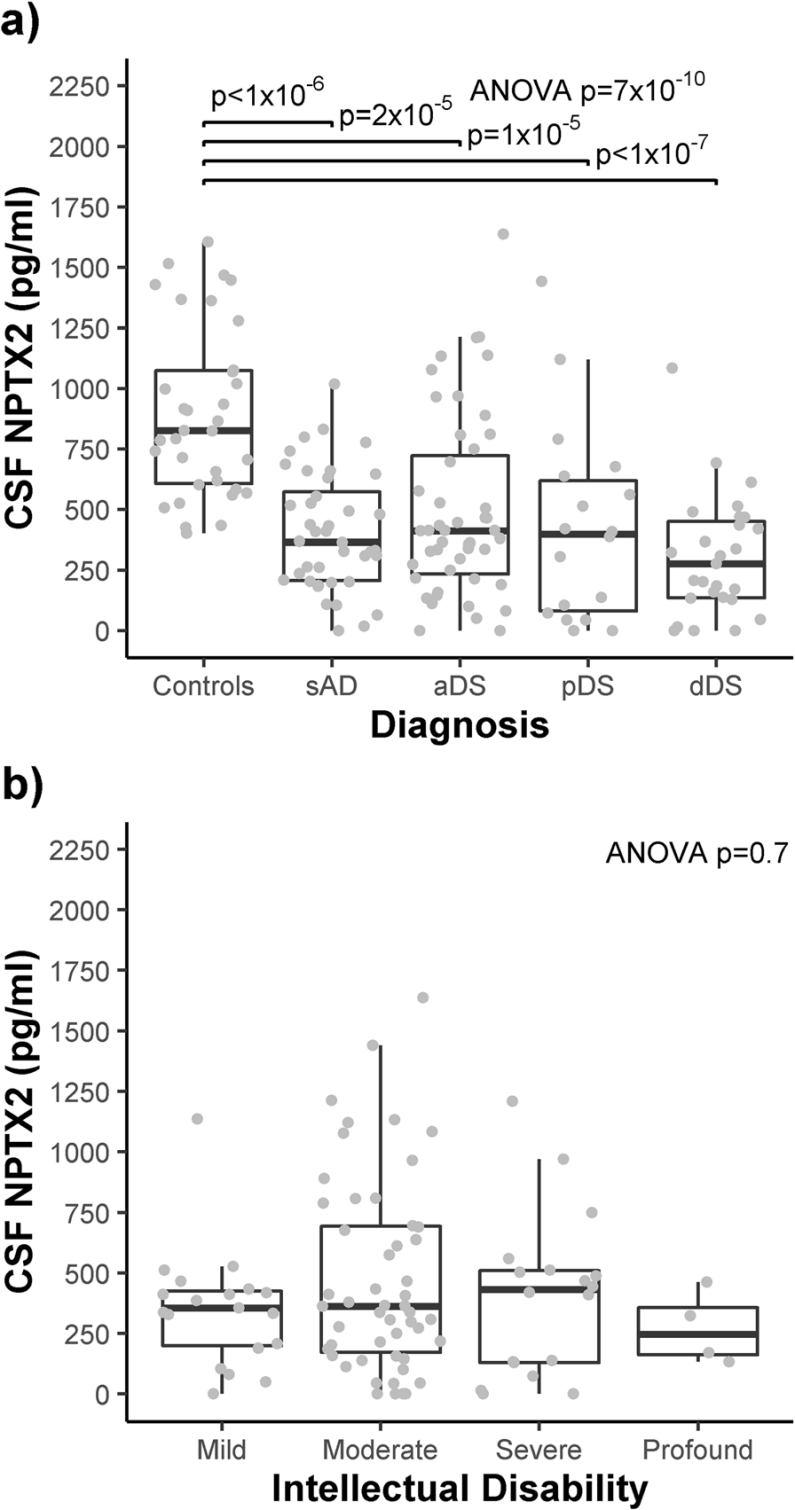
NPTX2 levels in control, sporadic AD and DS CSF. Box and whisker plots and individual points of the CSF levels of NPTX2 (pg/ml) are grouped (**a**) by clinical diagnosis and (**b**) by degree of intellectual disability (DS population only). *P*-values resulting from one-way ANOVA on square root transformed NPTX2 levels are shown in the top right corner. P-values resulting from Bonferroni-adjusted post-hoc Tukey test are shown where *p* < 0.05

Mean CSF NPTX2 levels were associated with clinical diagnosis (Fig. 1a; *n* = 168, *p* < 0.0001). Specifically, post hoc analyses showed that compared to controls, mean NPTX2 levels were 0.5-fold in sporadic AD (− 491 pg/ml, 95% CI −280 to − 702; adj.*p* < 0.0001), 0.6-fold in aDS (− 400 pg/ml, 95% CI − 196 to − 604; adj.*p* < 0.0001), 0.5-fold in pDS (− 472, 95% CI − 208 to − 736; adj.*p* < 0.0001) and 0.3-fold in dDS (− 595, 95% CI − 361 to − 828; adj.*p* < 0.0001). Mean NPTX2 levels were comparable between sporadic AD and DS (all AD stages adj.*p* > 0.3) and across AD stages in DS (all adj.*p* > 0.07).

In adults with DS, CSF NPTX2 levels were not associated with level of intellectual disability (Fig. 1b; *n* = 94, *p* = 0.7) and were not associated with any measure of cognitive performance in DS (*n* = 57–64, all *p* > 0.07). Mean NPTX2 levels were not associated with age overall (*p* = 0.6) or at any AD stage (all *p* > 0.1).

### Low CSF NPTX2 levels correlate with low CSF levels of a PV-interneuron specific receptor

**Fig. 2:**
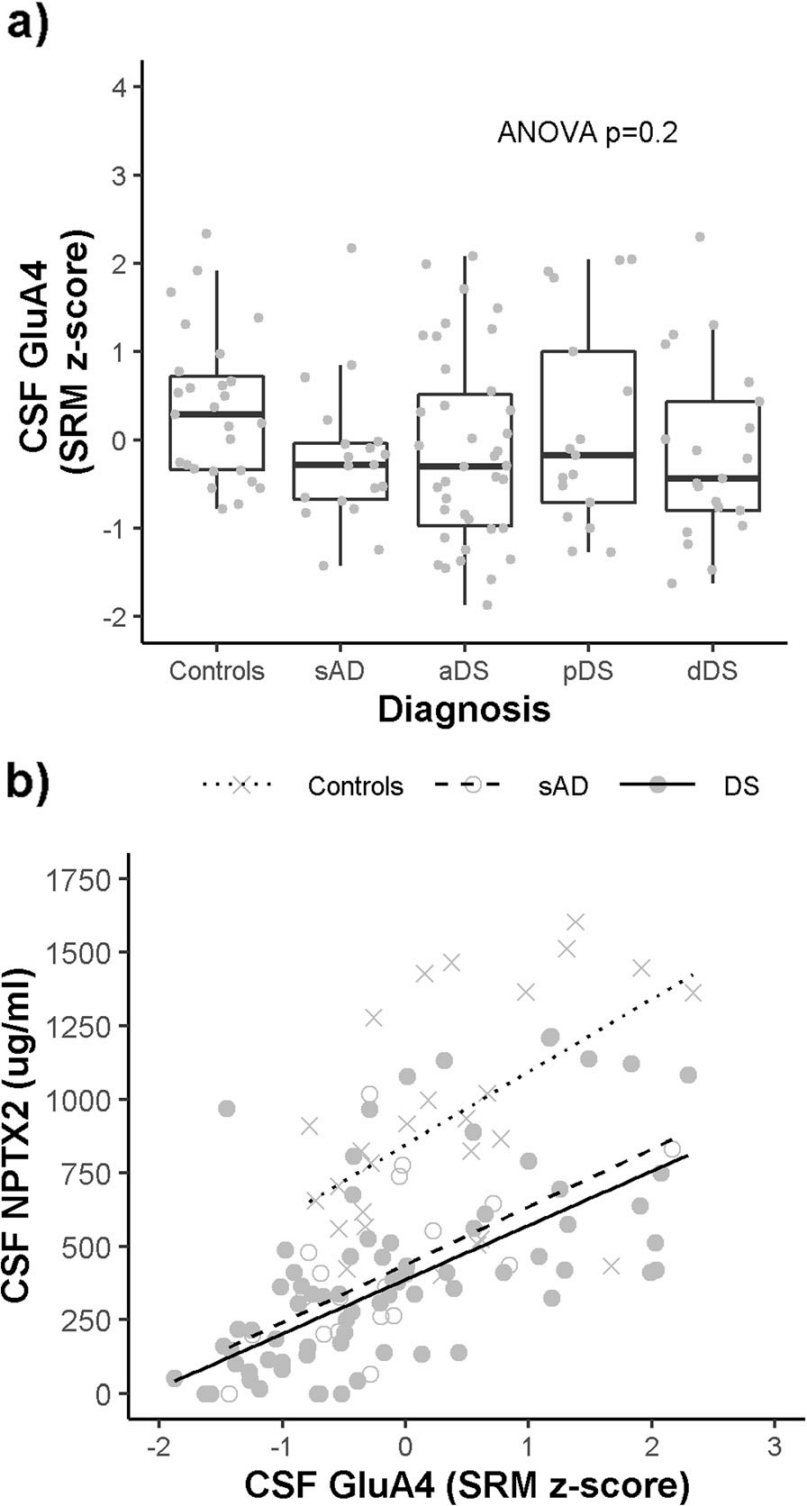
GluA4 levels in control, sporadic AD and DS CSF. **a** Box and whisker plots and individual points of the CSF levels of GluA4 (log2 intensity) grouped by clinical diagnosis. P-value resulting from one-way ANOVA on square root transformed GluA4 levels is shown in the top right corner. **b** CSF NPTX2 levels are plotted against CSF GluA4 levels with associated linear regression lines shown for controls, sporadic AD and DS groups (see legend)

We next sought to determine whether the PV-interneuron specific marker, GluA4, shows a coordinated reduction with NPTX2 in the CSF. While CSF GluA4 levels were not associated with clinical diagnosis (Fig. 2a; *p* = 0.2), low NPTX2 levels correlated with low GluA4 levels in controls (*n* = 27 *r*^2^ = 0.2, *p* = 0.003), sAD (*n* = 20, *r*^2^ = 0.4, *p* < 0.0001) and in DS (*n* = 81, *r*^2^ = 0.4, *p* < 0.0001): The association in DS was apparent at all AD stages; aDS (*n* = 36, *r*^2^ = 0.5, *p* = 0.04), pDS (*n* = 18, *r*^2^ = 0.5, *p* = 0.005) and dDS (*n* = 21, *r*^2^ = 0.4, *p* = 0.02).

### Low CSF NPTX2 levels correlate with CSF markers of AD pathology in adults with DS

**Fig. 3:**
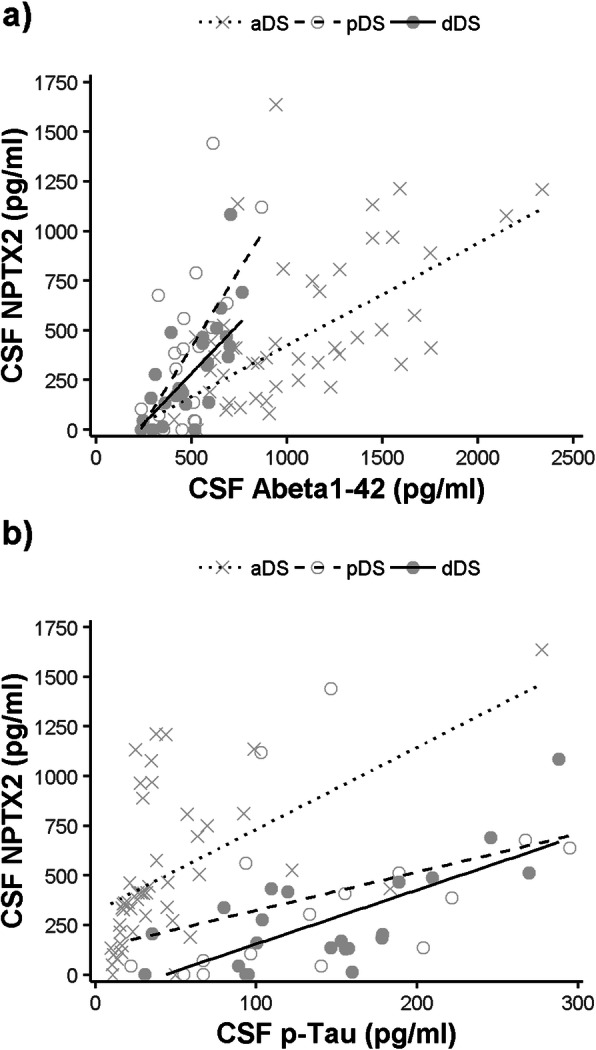
Correlation between CSF NPTX2 levels and CSF markers of AD pathology. CSF NPTX2 levels are plotted against (**a**) CSF Aβ_1–42_ and (**b**) CSF p-tau with associated linear regression lines shown for each clinical diagnosis (see legend)

We next sought to determine the relationship between CSF NPTX2 levels and core CSF AD biomarkers in DS. Low NPTX2 levels were associated with low CSF Aβ_1–42_ levels (Fig. 3a) in all diagnostic groups; aDS (*r*^2^ = 0.5, *p* < 0.0001), pDS (*r*^2^ = 0.3, *p* = 0.0003) and dDS (*r*^2^ = 0.6, *p* = 0.006). Low NPTX2 levels were also associated with p-tau (Fig. 3b) in all diagnostic groups; aDS (*r*^2^ = 0.4, *p* < 0.0001), pDS (*r*^2^ = 0.3, *p* = 0.0004) and dDS (*r*^2^ = 0.3, *p* = 0.001). Similar correlations were observed for CSF t-tau (*r*2 = 0.3 to 0.4, *p* < 0.003).

### Low CSF NPTX2 levels in adults with DS are associated with cortical atrophy and neuronal dysfunction in signature AD brain regions

**Fig. 4:**
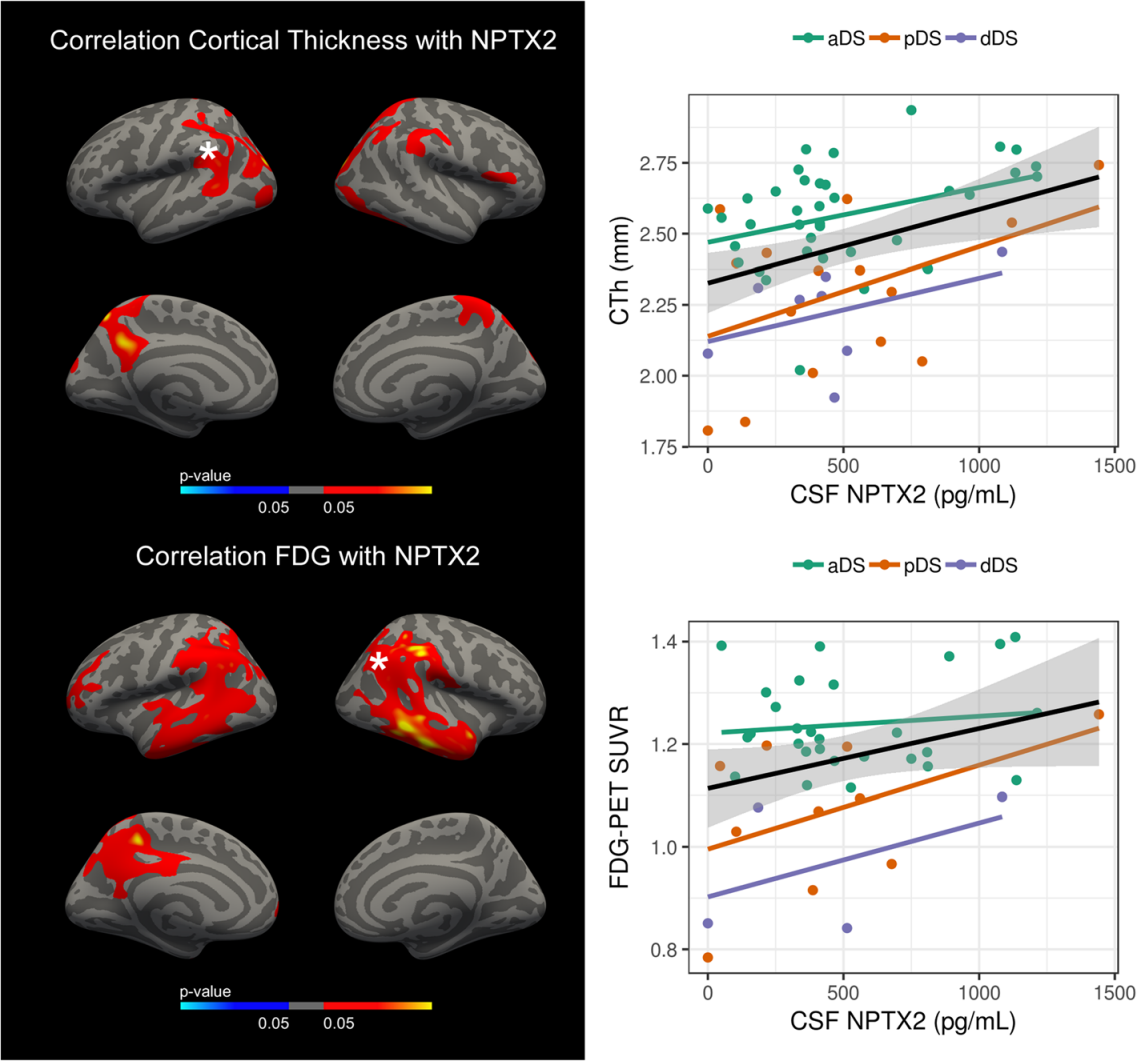
Structural and functional neuroimaging correlates of CSF NPTX2 levels in DS. Correlation of CSF NPTX2 levels and cortical thickness (top) and glucose metabolism (bottom) in all DS participants. Regions in red represent significant correlations (*p* < 0.05). Only the clusters that survived family-wise error correction *p* < 0.05 are shown. Scatterplots with linear regression line (black) and standard error (gray) show the correlation between CSF NPTX2 and neuroimaging biomarkers by diagnosis in a representative cluster that survived multiple comparisons, indicated with an asterisk. Regression lines are shown for each clinical group for informative purposes (see legend)

We also analyzed the relationship between CSF NPTX2 levels and structural and functional brain changes in adults with DS. As shown in Fig. 4, low CSF NPTX2 levels were associated with reduced cortical thickness in the temporal and parietal cortices (**top**; *n* = 63, FWE < 0.05) and with reduced glucose metabolism in more widespread regions of the temporal and parietal cortices (**bottom**; *n* = 44, FWE < 0.05).

## Discussion

Neuronal Pentraxin Receptor 2 (NPTX2) has recently been identified as a novel CSF biomarker of inhibitory circuit dysfunction in sporadic AD [12, 15]. Here we report that CSF levels of NPTX2 are also reduced in CSF of adults with DS who are highly susceptible to developing AD due to triplication at chromosome 21. The reduction in CSF NPTX2 compared to non-trisomic cognitively normal controls was observed in DS across all AD stages and was similar to the reduction observed in sporadic AD patientsboth here and in previous studies [12, 15]. This finding is consistent with studies that report a similar reduction in NPTX2 expression in postmortem sporadic AD and DS brains [12] and indicates that the mechanism underlying these changes is common to both sporadic and genetic forms of AD and that these changes may be reflected in the CSF. As these changes were apparent across all AD stages, CSF NPTX2 may be a useful marker of AD-related changes in adults with DS even prior to symptom onset.

DS cohorts with available CSF are scarce and current biofluid markers for AD in the DS population are restricted to surrogate markers of amyloidosis (CSF Aβ_1–42_ or amyloid PET tracers) and tau-mediated neurodegeneration (CSF p-tau and t-tau) combined with neuropsychological assessment, which can be confounded by substantial inter-individual variation in intellectual disability. NPTX2 represents a new addition to the DS biomarker arsenal that may be used to detect early signs of AD in this relatively under-studied population. Furthermore, this work opens the door to future studies exploring the potential of CSF NPTX2 as a prognostic marker for late-onset myoclonic epilepsy in Down syndrome and sporadic AD.

The lack of association of NPTX2 levels with intellectual disability suggests that the reduction in NPTX2 levels is not neurodevelopmental. This is supported by the finding that reduced CSF NPTX2 levels were associated with increased cortical atrophy and reduced glucose metabolism in temporal and parietal regions of the brain, regions that are particularly susceptible to AD pathology. Thus, CSF NPTX2 levels inversely correlated with the extent of both neuronal loss and neuronal dysfunction in signature AD regions. Taken together, we propose that changes in CSF NPTX2 levels in adults with DS are related to AD pathophysiology and not to intellectual disability.

Low CSF NPTX2 levels in adults with DS were associated with low CSF GluA4 and low CSF Aβ_1–42_, a surrogate CSF marker of increased aggregation of brain Aβ_1–42_. This is the first evidence that a synergized increase in brain Aβ_1–42_ aggregates and reduced NPTX2 can be measured indirectly in the CSF of adults with DS. NPTX2 is expressed by pyramidal neurons and secreted by axon terminals to mediate activity-dependent strengthening of pyramidal neuron excitatory synapses on GABA-ergic PV interneurons, a mechanism that is dependent upon the AMPA receptor subunit, GluA4 [11]. Histological studies have shown reduced NPTX2 and GluA4 expression in signature AD regions of AD and DS brains [12], regions that also show a reduced number of PV interneurons [37]. This is consistent with the accumulating evidence for reduced inhibitory interneuronal activity in the AD brain [10]. Studies in mice [37–39] have shown that low NPTX2 expression at excitatory synapses of PV interneurons combined with brain amyloidosis is critical for inhibitory circuit dysfunction [12].Thus, a hypothesis emerges whereby a loss of NPTX2-GluA4 synapses in regions affected by amyloidosis may lead to inhibitory circuit dysfunction in AD. While extrapolation of data from mouse to human brain should be treated with caution, this could be one plausible explanation for the low CSF NPTX2 levels we report in sporadic AD patients and adults with DS.

Unlike NPTX2, GluA4 levels were not significantly reduced in CSF of adults with DS compared to controls, making NPTX2 a potentially more valuable CSF biomarker in DS. Based on these findings, CSF NPTX2 can be added to the growing list of biomarkers that show changes similar, both in direction and magnitude, to those described in sporadic or autosomal dominant AD [4, 20, 40]. The potential of CSF NPTX2 as a novel biomarker of interneuronal dysfunction can now be extended to DS, a genetic form of AD that targets the same cortical regions as the sporadic and autosomal dominant forms [5].

CSF Aβ_1–42_, t-tau and p-tau are excellent diagnostic markers in sporadic AD and in the DS population and we do not expect NPTX2 to improve upon those markers. Rather, this study supports the increasing literature that suggests CSF NPTX2 as a surrogate marker of underlying interneuronal circuit dysfunction in sporadic and, now genetic, forms of AD even at the preclinical stage. Further studies in longitudinal cohorts are needed to explore the full potential of CSF NPTX2. The capacity of NPTX2 as an early prognostic marker would be one potential use worth exploring for example. Alternatively, as tau and Aβ are common drug targets in AD clinical trials, a surrogate measure of synaptic dysfunction that does not directly measure the drug target levels would be a useful addition to the biomarker arsenal.

In previous studies in sporadic AD, CSF NPTX2 levels correlated better with cognitive performance than the core AD biomarkers [12]. Furthermore, in genetic frontotemporal dementia, low CSF NPTX2 levels predicted subsequent decline in phonemic verbal fluency and Clinical Dementia Rating scale [41]. In contrast, here we report that reduced CSF NPTX2 levels were not associated with any measure of cognitive performance in adults with DS. That being said, the high variability in intellectual disability in the DS population can complicate cognitive assessment and therefore these neuropsychological tests could only be performed in individuals with mild or moderate intellectual disability, which resulted in a limited sample size. Thus, the potential relationship between CSF NPTX2 levels and cognitive performance in the DS population warrants further study in a larger population. On the other hand, there is evidence that the temporal onset of cognitive decline may differ in DS compared to autosomal dominant AD [4], thus underscoring the importance of considering the neurodevelopmental differences in the DS population when interpreting the cognitive results.

The association of increased CSF NPTX2 levels with increased CSF p-tau levels in the DS population could indicate that widespread neuronal loss at later disease stages may lead to increased NPTX2 clearance into the CSF and may mask a reduction in NPTX2 synapses. Nevertheless, the inverse relationship we report between CSF NPTX2 levels and a neuroimaging marker of cortical atrophy, suggests that neurodegeneration has a discernable but limited confounding effect on CSF NPTX2 levels.

A limitation of this study is the cross-sectional design; longitudinal studies are needed to fully establish the prognostic value of NPTX2 in both sporadic AD and in DS. Furthermore, these findings warrant replication in an independent DS cohort. Exploration of the potential of NPTX2 as a blood biomarker would also be valuable to minimize the use of lumbar puncture in people with DS.

## Conclusions

Here we report that CSF NPTX2 levels are reduced in adults with DS compared to cognitively normal, non-trisomic controls even prior to AD onset. Similar reductions were observed in the CSF of sporadic AD patients and have been previously reported in postmortem AD and DS brains [12, 15]. Substantial evidence from animal studies suggests that such changes may be associated with reduced inhibitory inter-neuronal activity. While further functional studies are needed, this could explain the correlation we report between low CSF NPTX2 and low CSF GluA4, the predominant AMPA receptor subunit in PV interneurons. In conclusion, this study shows for the first time that NPTX2 could be a valuable CSF biomarker of AD-related inhibitory neuronal circuit dysfunction in adults with DS even prior to symptom onset.

## Data Availability

The data analyzed during the current study are available from the corresponding author on reasonable request.

## Abbreviations

AD: Alzheimer’s disease
aDS: Asymptomatic AD in the context of DS
CAMCOG: Cambridge Cognition Examination
CSF: Cerebrospinal fluid
DABNI: Down Alzheimer Barcelona Neuroimaging Initiative
dDS: AD dementia in the context of DS
DS: Down syndrome
FDG: [^18^F]-fluorodeoxyglucose
FWE: Family wise error
FWHM: Full width at half maximum
GABA: Gamma-Aminobutyric acid
MRI: Magnetic resonance imaging
NPTX2: Neuronal pentraxin-2
pDS: Prodromal AD in the context of DS
PET: Positron emission tompgraphy
PV: Parvalbumin
SPIN: Sant Pau Initiative for Neurodegeneration
SRM: Selected Reaction Monitoring
